# P-20. Estimating the Public Health Impact of Pertussis Decennial Booster Vaccination for German Adults

**DOI:** 10.1093/ofid/ofae631.228

**Published:** 2025-01-29

**Authors:** Victoria G Soares, Maria Waize, Pavo Marijic, Alexander Heiseke, Daniel Heinze, Nicolas Jamet, Hiral Shah, Pavitra Deeywadaa, Jorge Jacob, Stavros Oikonomou, Frederik Verelst, Eliana Biundo

**Affiliations:** GSK, Wavre, Brabant Wallon, Belgium; GSK, Wavre, Brabant Wallon, Belgium; GSK, Wavre, Brabant Wallon, Belgium; GSK, Wavre, Brabant Wallon, Belgium; GSK, Wavre, Brabant Wallon, Belgium; GSK, Wavre, Brabant Wallon, Belgium; GSK, Wavre, Brabant Wallon, Belgium; GSK, Wavre, Brabant Wallon, Belgium; IQVIA, London, England, United Kingdom; IQVIA, London, England, United Kingdom; GSK, Wavre, Brabant Wallon, Belgium; GSK, Wavre, Brabant Wallon, Belgium

## Abstract

**Background:**

Pertussis is a highly contagious respiratory disease affecting both children and adults. Current vaccination strategies primarily target infants, young children, and pregnant women, shifting the disease burden towards adults, with age and comorbidities increasing the risk of severe disease. This study aims to estimate the potential public health impact of decennial booster vaccination in adults in Germany.

**Methods:**

A dynamic transmission model (DTM) was developed to evaluate the public health impact of alternative strategies for pertussis booster vaccination in adults (≥ 18 years) and those at increased risk. Disease transmission dynamics occurred at total population level with high-risk and non-high-risk populations tracked in separate compartments. The DTM was populated with published German data sources for population, vaccination coverage and incidence. Incidence was adjusted with previously published under-reporting rates. The model evaluated the age-stratified incidence and impact on healthcare resource utilization over a 100-year horizon. As base case, a decennial booster strategy for all adults was assessed in replacement of the current recommendation of a single adult (≥ 18 years) booster. Vaccination strategies targeting only high-risk populations were tested in scenario analyses.

**Results:**

Interim results for the decennial booster strategy in adults suggest that administering an incremental 171 million booster doses would prevent over 35 million symptomatic cases, 1.7 million hospitalizations and 215,000 cases with complications, over 100 years. The model predicts that 5 individuals need to be vaccinated to avoid a pertussis case and 100 to avoid hospitalization. At equilibrium, the population overall incidence (symptomatic and asymptomatic) is expected to decrease from around 1,480 cases to 930 cases (-38%) per 100,000 person-years.

**Conclusion:**

Modelling results suggest that implementing adult decennial booster vaccinations can significantly alleviate the burden of pertussis in Germany.

**Disclosures:**

**Victoria G. Soares, MBA**, GSK: Advisor/consultant **Maria Waize, MSc**, GSK: Employed by GSK **Pavo Marijic, PhD**, GSK: Employed by GSK **Alexander Heiseke, PhD**, GSK: Employed by GSK|GSK: Stocks/Bonds (Public Company) **Daniel Heinze, PhD**, GSK: Employed by GSK|GSK: Stocks/Bonds (Public Company) **Nicolas Jamet, PharmD**, GSK: Employed by GSK **Hiral Shah, PhD**, GSK: Employee of GSK|GSK: Stocks/Bonds (Public Company) **Pavitra Deeywadaa, MBBS, MD**, GSK: Employed by GSK|GSK: Stocks/Bonds (Private Company) **Frederik Verelst, PhD**, GSK: Employed by GSK|GSK: Stocks/Bonds (Public Company) **Eliana Biundo, MSc**, GSK: Employee|GSK: Stocks/Bonds (Private Company)

